# Elective cricothyrotomy in a dog with transient laryngeal paralysis secondary to Australian paralysis tick (*Ixodes holocyclus*) envenomation

**DOI:** 10.1111/avj.13175

**Published:** 2022-05-26

**Authors:** S Hardjo, KJ Nash, SK Day, MD Haworth

**Affiliations:** ^1^ UQ VETS, School of Veterinary Science The University of Queensland Gatton Queensland

**Keywords:** airway, cricothyrotomy, laryngeal paralysis, tick paralysis, tracheostomy

## Abstract

The tube cricothyrotomy (CTT) has recently been introduced to small animal medicine as a viable surgical airway access procedure; however, there are no reports documenting its clinical use. The author's objective is to describe the clinical application, complications, and management of an elective CTT in a dog. Furthermore, the characteristics of CTT that may be clinically advantageous over temporary tube tracheostomy (TT) will be discussed. A 2‐year‐old female spayed German shepherd dog required mechanical ventilation (MV) due to unsustainable work of breathing as a result of tick paralysis and aspiration pneumonia. After successful weaning from MV, the dog was diagnosed with laryngeal paralysis. A surgical airway was performed using CTT to allow extubation and patient management whilst conscious. Complications included frequent tube suctioning due to accumulation of airway secretions in the tube and a single dislodgement event. The dog made an uneventful recovery with complete stoma healing by the second intention within 15 days. To the authors' knowledge, this is the first clinical report of an elective CTT performed to successfully manage upper airway obstruction in the dog. Its efficacy, clinical management and patient outcome are described.

The elective tube cricothyrotomy (CTT) gained wider acceptance in human medicine following a landmark study in 1976, which demonstrated its safety and efficacy in 655 cases where tube tracheostomy (TT) would otherwise be indicated.^1^ In veterinary medicine, CTT has recently been suggested as a reasonable alternative to TT for emergency surgical airway access.^2^ Although successful CTT has been described in experimental studies using healthy dogs^3^, ^4^ to the authors' knowledge, there are no published peer‐reviewed reports of its use in clinical settings.

Dogs with *Ixodes holocyclus* induced tick paralysis suffer from a lower motor neuropathy that may also cause laryngeal paralysis as a result of the inhibition of acetylcholine release at the neuromuscular junction.^5^, ^6^ Experts in the field recommend a surgical airway using TT to manage laryngeal paralysis causing temporary upper airway obstruction secondary to tick paralysis.^7^, ^8^ The aim of this report is to describe the clinical management and outcome of a dog with laryngeal paralysis secondary to tick paralysis managed with elective CTT. A secondary aim is to discuss potential management advantages of CTT in light of the current standard of practice of TT for surgical airway access in veterinary medicine.

## Case summary

### Presentation

A 2‐year‐old female spayed German shepherd dog weighing 20.7 kg was referred to the emergency department for a 12‐h history of generalised weakness progressing to non‐ambulatory tetraparesis. A paralysis tick (*Ixodes holocyclus*) was removed by a veterinary nurse at the referring practice, two hours before the presentation. On the initial physical exam, the dog's neurologic and respiratory status was graded 3C.^9^ An increased respiratory effort was observed with harsh lung sounds and crackles auscultated, predominantly in the ventral lung fields bilaterally. The dog regurgitated during the initial exam and regurgitated fluid was noted to be exiting from both nares. Initial venous blood gas results at presentation demonstrated a mild acidemia, most consistent with respiratory acidosis, as seen in Table1A. Point‐of‐care thoracic ultrasound revealed 4–5 B‐lines per intercostal space in the cranioventral left lung fields and areas of coalescing B‐lines in the right middle and right cranial lung fields suggestive of extravascular lung fluid.^11^, ^12^ Three‐view thoracic radiographs performed shortly afterwards confirmed a ventrally dependent right middle and cranial lobe alveolar infiltrate, most consistent with aspiration pneumonitis/pneumonia.

**Table 1:** Blood gas parameters from a 2‐year‐old female spayed German shepherd dog, diagnosed with tick paralysis that underwent mechanical ventilation and temporary cricothyrotomy

	Reference intervals^10^	(A)	(B)	(C)	(D)
Parameters	Arterial/peripheral venous	V	A	A	A
FiO_2_ if arterial			0.6		0.4
pH	7.35–7.46/7.34–7.38	7.342	7.322	7.20	7.376
PCO_2_	32–43/40–46 mmHg	45.1	52.0	63.5	38.0
PO_2_	80–105/48–57 mmHg	84.7	169	444	142
Na^+^	140–150 mEq/L	147	145	146	150
Cl^−^	109–120 mEq/L	112	108	113	119
K+	3.9–4.9 mEq/L	4.0	3.6	4.5	4.5
iCa^2+^	1.25–1.5 mmol/L	1.30	1.26	1.33	1.35
Glucose	3.6–6.2 mmol/L	6.0	7.1	8.0	8.0
Lactate	0.5–2.0 mmol/L	1.2	0.8	0.5	0.4
Haematocrit	40.3%–60.3%	54.0			—
HCO_3_^−^	18–26/22–24 mmol/L	23.0	—	—	—
AG	8–21 mmol/L	10.9	10.3	8.6	8.0
Base excess	−1 to 5/−2 to 0 mmol/L	1.3	0.8	−3.6	−3.0

*Note*: (A) Venous blood gas values at the time of presentation to the hospital, (B) arterial blood gas values shortly after the commencement of the first mechanical ventilation event, (C) arterial blood gas values after the second mechanical ventilation event, (D) arterial blood gas values before weaning from second ventilation event.

### Treatment

A timeline of the key treatment events can be found in the Appendix A. One millilitre per kilogram of tick antiserum (AVSL Ixodes Holocyclus Antivenom, Ixodes Holocyclus Antivenom >500 units/mL, preservative: 0.0022 mL/mL phenol; Australian Veterinary Serum Laboratories, 20–22 Uralba Street, Lismore, NSW 2480) was administered intravenously over 20 min without complication. Over the following 3 h, the dog developed marked respiratory difficulty characterised by orthopnoea, abdominal breathing and intermittent gasping. The dog's work of breathing was assessed by a board‐certified criticalist to be unsustainable and the dog was at risk of imminent fatigue and respiratory arrest. This is one of four described indications^13^ for mechanical ventilation (MV) in dogs and cats with tick paralysis, hence, the dog was promptly intubated and commenced on MV. An arterial blood gas analysis revealed the ratio of partial pressure of arterial oxygen (PaO_2_) to the fraction of inspired oxygen (FiO_2_) was 282, consistent with mild acute respiratory distress syndrome^14^ (see Table 1B). The ventilator was initially set on volume‐controlled, assist control mode with 5.0 cm H_2_O positive end‐expiratory pressure and a tidal volume of 8 mL/kg. The respiratory rate was 20 breaths per minute with FiO_2_ reduced from 100% to 50% within the first hour. Medications initially used to facilitate MV included butorphanol (ilium Butorgesic. butorphanol [as tartrate], Troy laboratories PTY LTD, 37 Glendenning Road, Glendenning NSW 2761, Australia) 0.05–0.3 mg/kg/h, midazolam (Pharmaco Australia Ltd, Gordon NSW 2072, Australia) 0.1–0.5 mg/kg/h, propofol (B. Braun Australia Pty. Ltd., Level 5, 7–9 Irvine Pl, Bella Vista NSW 2153, Australia) 0.05–0.2 mg/kg/min. Initial empirical antibiotics were commenced with amoxicillin/clavulanic acid (Juno Pharmaceuticals Pty Ltd, Cremome VIC 3121, Australia) 25 mg/kg IV q8h. The ventilator mode was changed to synchronized intermittent mandatory ventilation with 8 cm H_2_O of pressure support three hours after the commencement of MV and ketamine (Ketamav 100, MavLab, Slacks Creek, QLD 4127, Australia) infusion was subsequently commenced at 5 mcg/kg/min to assist in maintaining adequate anaesthetic depth.

A routine spontaneous breathing trial was performed approximately 20 h after starting positive pressure ventilation, but the dog became rapidly hypercapnoeic based on end‐tidal CO_2_ and MV was restarted. A second spontaneous breathing trial was attempted 36 h after commencing ventilation when the dog demonstrated regular spontaneous breathing efforts and the ventilator mode was changed to spontaneous with pressure support. The pressure support was reduced to 3 cm H_2_O over two hours and the dog was successfully weaned from MV and extubated.

The dog's respiratory effort began to increase markedly with audible stridor at 18 h after extubation. Medetomidine (ilium Medetomidine: 1 mg/mL medetomidine hydrochloride, Troy laboratories PTY LTD, 37 Glendenning Road, Glendenning NSW 2761, Australia) 2 mcg/kg was given IV once for sedation and to facilitate upper airway suctioning and examination. The dog was concurrently receiving a medetomidine constant rate infusion (CRI) at 2 mcg/kg/h and butorphanol CRI at 0.18 mg/kg/h. Paradoxical medial movement of the arytenoid cartilages was observed during inspiration, and the dog was diagnosed with laryngeal paralysis. The dog was re‐intubated and the PaCO_2_ 5 min following intubation was 63.5 mm Hg (Table 1C), suggesting ventilatory failure. Mechanical ventilation was then recommenced to manage recurrent ventilatory failure.

### Cricothyrotomy

Eight hours following the commencement of the second MV event, oxygenation and ventilation parameters were deemed satisfactory while receiving MV in spontaneous mode with pressure support (see Table 1D). In order to assist with weaning and extubation, a CTT was performed at the bedside in the intensive care unit by a third‐year resident in emergency and critical care, based on a recently described open surgical method.^15^

The patient was positioned in dorsal recumbency with a towel between the dorsal neck and the table.

A 20 cm × 10 cm area of hair was shaved from the ventral neck and the skin was aseptically prepared. A 5 cm skin incision was made on the midline and centred over the cricothyroid membrane. The sternohyoid muscle was separated using a combination of sharp and blunt dissection. Haemostasis was achieved with Kelly haemostats.

An incision was made over the length of the cricothyroid ligament with a #15 blade, entering the airway and the endotracheal tube was removed. Purulent material was visible around the external surface of the endotracheal tube and within the airway. An 8.0 mm internal diameter/11.0 mm outer diameter tracheostomy tube (SurgiVet™ Cuffed Silicone Tracheostomy Tubes [V60080], 8 mm I.D. 8.8 cm Long, Smiths Medical ASD, Inc, St. Paul, MN 55112 USA) was placed through the cricothyrotomy incision. The tube cuff was inflated, and the patient was reconnected to the ventilator via the CTT tube. A stay suture was placed around the ventral cricoid ring using 0 PDS (PDS*II [Polydioxanone Suture], Ethicon, INC 2007, Johnson & Johnson Medical Pty Ltd (Australia), 1–5 Khartoum Road, North Ryde, NSW, 2113, Australia). The tube was secured around the neck using non‐adhesive cotton tape (12 mm cotton tying tape, Archival survival, Doncaster East Vic 3109, Australia). The dog was subsequently weaned slowly from MV, recovering uneventfully with the CTT *in situ*. The second ventilation event lasted for approximately 18 h.

The incision was not partially closed during the initial surgery to assess if the mobility of the larynx would obscure the stoma. During tube management, the incision size appeared more than adequate, and the proximal portion was subsequently closed with three interrupted sutures using 2–0 nylon (Sharpoint Nylon Black Monofilament. Precision reverse cutting. Icon Medical Supplies, B/75–77 St Hilliers Rd, Auburn NSW 2144, Australia).

### Cricothyrotomy management

The CTT tube was managed with our hospital's protocol for TT care. This included nebulisation through the tube for hydrating airway secretions, followed by tube suctioning using a flexible suction tip with vacuum control (Y‐suction catheter with two eyes, FG 10 cm × 43 cm, ConvaTec Ltd., Deeside, CH5 2NU, UK). Tube suctioning was initially performed as required due to the accumulation of respiratory secretions in the tube and this averaged once every 2 h for the first 12 h, including one complete tube change. Following this initial management, nebulisation and tube suctioning were performed routinely at four‐hour intervals. Stoma site cleaning with dilute 0.05% chlorhexidine solution and tube changes were performed routinely every 8 h.

**Figure 1:**
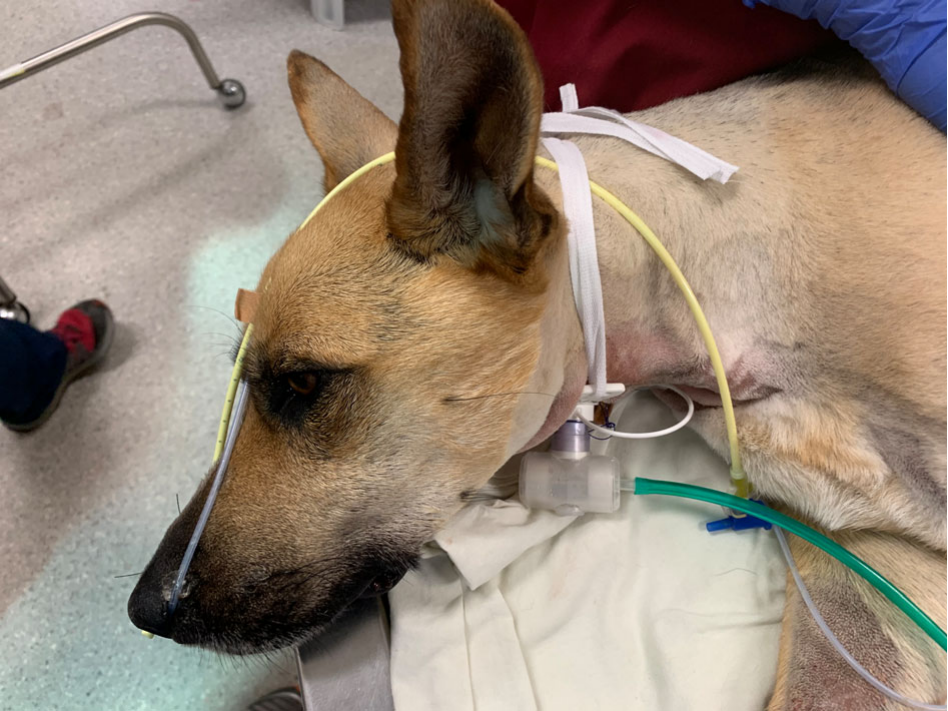
A 2‐year‐old female spayed German shepherd dog diagnosed with laryngeal paralysis secondary to tick paralysis. A cricothyrotomy tube has been placed for airway management and is well tolerated. An heat and moisture exchange filter in a T‐tube design with oxygen supplementation is attached to the cricothyrotomy tube. A nasogastric feeding tube has been placed through the right nare and an oxygen catheter in the left (not in use).

Oxygen supplementation was initially administered at 3 L/min via a designated port on a heat and moisture exchange filter (T type Heat and Moisture Exchanger Trach, Vincent Medical manufacturing Co., Ltd., Flat/RM B2, 7/F., Hang Fung Industrial Building, Phase 2, 2G Hok Yuen Street, Hung Hom, Kowloon, Hong Kong) and continued via a nasal catheter after tube removal as per Figure 1. Oxygen supplementation was discontinued approximately 48 h following the cessation of positive pressure ventilation.

**Figure 2:**
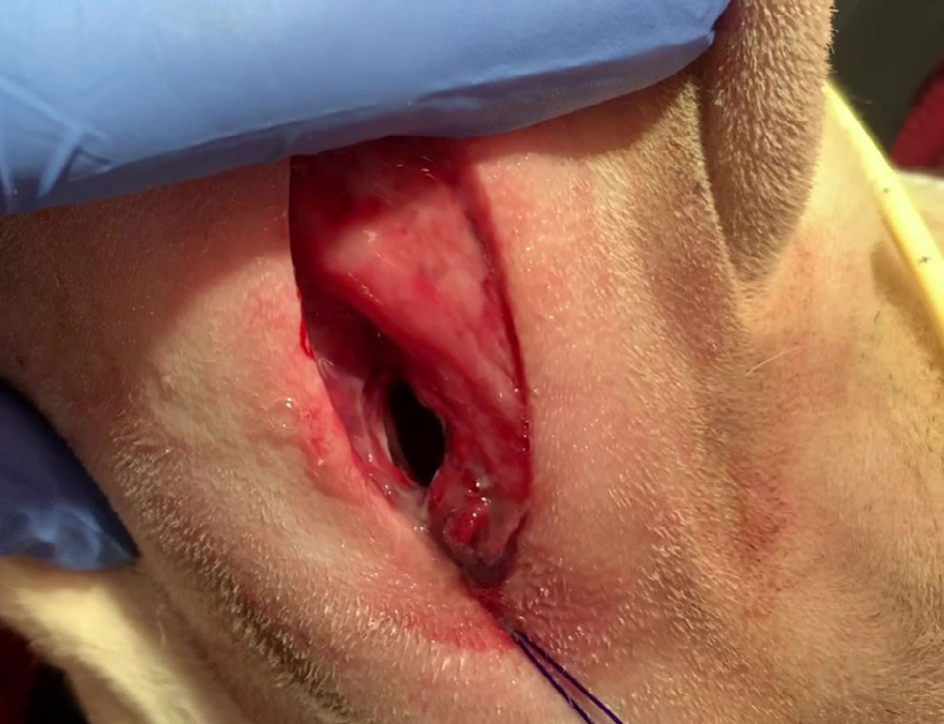
A 2‐year‐old female spayed German shepherd dog diagnosed with laryngeal paralysis secondary to tick paralysis had a cricothyrotomy performed for airway management following mechanical ventilation. The cricothyrotomy stoma was readily apparent with retraction of the skin alone and allowed good airflow without the tube. The stay suture can be retracted distally to stabilize the larynx for tube changes, as seen in this photo.

A single stay suture had been placed with the intention to assist locating the stoma with ventral retraction, but this was rarely required as the superficial location of the larynx made the stoma easy to visualise. It was sometimes used to stabilize the larynx with distal retraction for tube introduction. It was noted that there was good airflow through the stoma with retraction of the skin alone, as per Figure 2.

A single episode of tube dislodgement occurred on day 4 while changing the patient's recumbency. The partially dislodged tube was readvanced by a veterinary technician without removing it completely, and this immediately resulted in signs of obstruction. The tube was found to be directed proximally, towards the glottis, and was removed. This resulted in good airflow through the stoma and immediate resolution of respiratory difficulty. All staff involved in tube replacement noted the procedure to be very straightforward and there were no challenges locating the stoma. The tube CTT was well tolerated by the dog while awake and no episodes of coughing or irritation were noted (see Figure 1).

After CTT tube maintenance for approximately 36 h, the dog's ability to breathe through the upper airway was assessed as normal by removing the tube and covering the stoma with a sterile gloved finger for 30 s. The CTT tube was not replaced, and no signs of upper respiratory tract obstruction were seen. Antibiotics were continued orally with amoxicillin/clavulanic acid (Dechra Veterinary Products (Australia) Pty Ltd, 2 Cal Close, Somersby NSW 2250, Australia) at 22 mg/kg PO q12h and the dog was discharged seven days after presentation. Oral antibiotics were continued for a further eight days with patient reassessments at one and two weeks following discharge.

### Healing

**Figure 3:**
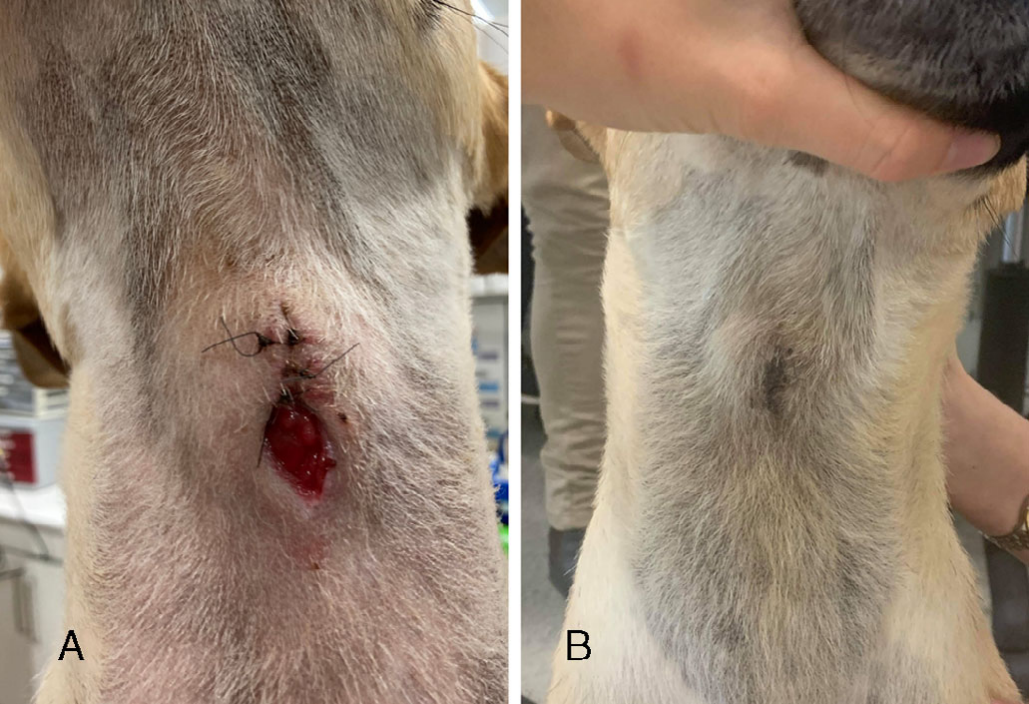
A 2‐year‐old female spayed German shepherd dog diagnosed with laryngeal paralysis secondary to tick paralysis had a temporary tube cricothyrotomy procedure for airway management following mechanical ventilation. This photo demonstrates healing of the cricothyrotomy stoma at seven days (A) and at 15 days **(**B) post tube removal.

The CTT stoma was left to heal by second intention and seven days following tube removal, granulation tissue had completely covered the stoma (see Figure 3A). The respiratory character was normal at this time but there were episodes of coughing two to three times per day. At 15 days following tube removal, the wound had epithelialized and healed routinely (see Figure 3B). All coughing had ceased.

### Follow up

At 42 days following tube removal, the dog had no further coughing, no changes in phonation, normal exercise tolerance and no noticeable wound.

## Discussion

Laryngeal paralysis is a common sequela to tick paralysis and TT is recommended for airway management.^8^ The diagnosis was made after the patient was administered medetomidine and butorphanol sedation, at doses that should not affect laryngeal function, based on studies using combinations of its active constituent isomer, dexmedetomidine with butorphanol.^16^ Performance of a surgical airway in cases of functional laryngeal compromise from tick paralysis is beneficial for weaning from MV and conscious management of patients.^8^ This negates the need for an otherwise unnecessary prolonged general anaesthesia in dogs with adequate oxygenation and ventilation, reduces costs, and reduces clinical resources associated with the higher level of intensive care for orotracheally intubated patients.

The recommended surgical airway procedure to bypass upper airway obstructions in small animal veterinary medicine has traditionally been the TT.^17^ Elective CTT has been performed as an alternative for temporary surgical airway access in human medicine^1^ and there may be advantages for performing it in the dog. Due to the proximal and superficial location of the larynx in the cervical neck, there is an extremely low chance of left recurrent laryngeal nerve or major blood vessel damage during the CTT procedure when compared to the TT. The anatomy is easily palpated and visualised, and there are fewer steps, making the procedure less technically challenging and quicker than the TT.^2^ Given the described benefits and lack of contraindications, in this case, elective CTT was chosen as a reasonable alternative to TT. There were no complications encountered when performing the CTT procedure, apart from minor and self‐limiting bleeding from the sternohyoid muscle during the initial procedure.

In one study, the most common complications observed in the management of TT (in descending frequency) were obstruction of the tube, dislodgment of the tube, and aspiration pneumonia.^18^ Obstruction of the tube with airway secretions was noted in this case, requiring frequent suctioning during initial management. However, this was likely largely due to the previously diagnosed aspiration pneumonia, with evidence of copious airway secretions present at the time of CTT placement. Furthermore, the frequency of tube suctioning reduced over time, indicating the CTT itself was unlikely to be the causative factor for this complication.

There are several advantageous anatomical features of the ventral larynx in the dog that can potentially mitigate complications and improve tube management in the veterinary intensive care unit when compared to the TT. Partial tube dislodgement was observed once in this case and full dislodgement was likely avoided due to the characteristics of the cricothyroid space. The complete ring of the cricoid cartilage with its rostral protrusion and superficial position in the neck potentially offers additional support against tube dislodgement.

Immediate tube removal to establish a patent airway during the obstructive event, in this case, demonstrates another advantage of the laryngeal anatomy in the dog. Transverse or flap tracheal incisions utilised in TT allows the tracheal rings to readily recoil to their original position following tube removal, which may contribute to obstruction of the stoma.^19^ On the other hand, the CTT produces a stoma surrounded by firm cartilages allowing it to remain open without a tube. This is also aided by the region of the neck targeted by CTT, as soft tissue depth and skin folds are relatively minimised in this area when compared with the TT.^2^, ^18^

Tracheostomy stomas may be difficult to locate and usually require appropriately labelled stay sutures in the tracheal rings immediately proximal and distal to the tracheal incision.^19^, ^20^ These sutures need to be correctly identified immediately in the event of unexpected dislodgement or emergent tube change due to obstruction. Retraction of these stay sutures in the correct directions is required before tube insertion and can be challenging particularly for a single operator. Given the superficial nature of the CTT stoma, it can be located rapidly and only one stay suture is required, reducing confusion and the steps required for tube changes. Stoma exposure and re‐intubation could often be performed without the use of the stay suture in this case.

## Limitations

While direct comparisons cannot be made to TT in this single case report, the authors document the presence of clinical characteristics of the CTT procedure and tube management that are potentially beneficial. Prospective studies with patients randomised to receive temporary TT or CTT are required to objectively compare complications and outcomes between procedures. The findings in this case report cannot be generalised to all dogs with all forms of upper airway obstruction.

## Conclusion

Elective CTT was successfully implemented in this case of a dog with laryngeal paralysis secondary to tick paralysis. There were numerous perceived benefits over the authors' experience with TT including ease of stoma location, stoma patency without a tube and possible resistance to dislodgement. The procedure had fewer steps and was technically simpler than TT, which may allow for a greater proportion of veterinarians to perform it quickly at the bedside.

This is the first reported use of CTT in a clinical canine patient. This report describes the efficacy of CTT as a temporary surgical airway and details the uneventful recovery of the patient. Elective CTT can be considered as a reasonable alternative to TT.

## Conflicts of Interest/Funding Information

The authors declare no conflicts of interest or sources of funding for the work presented here.

## Appendix A

*Note*: Timeline of key events during hospitalisation of a 2‐year‐old female spayed German shepherd dog hospitalised for tick paralysis. cm H_2_O, centimetres of water; CTT, Cricothyrotomy; MV, mechanical ventilation; RR, Respiratory rate; SPO_2_, Saturation of arterial haemoglobin via pulse oximetry; TAS, Tick anteserum.
